# Knowledge, attitudes and other factors associated with assessment of tobacco smoking among pregnant Aboriginal women by health care providers: a cross-sectional survey

**DOI:** 10.1186/1471-2458-12-165

**Published:** 2012-03-07

**Authors:** Megan E Passey, Catherine A D'Este, Robert W Sanson-Fisher

**Affiliations:** 1 University Centre for Rural Health-North Coast, School of Public Health, University of Sydney, PO Box 3074, Lismore NSW 2480, Australia; 2 School of Medicine and Public Health, Faculty of Health, University of Newcastle, David Maddison Building, King St, Newcastle NSW 2300, Australia

## Abstract

**Background:**

As with many Indigenous peoples, smoking rates among Aboriginal Australians are considerably higher than those of the non-Indigenous population. Approximately 50% of Indigenous women smoke during pregnancy, a time when women are more motivated to quit. Antenatal care providers are potentially important change agents for reducing the harms associated with smoking, yet little is known about their knowledge, attitudes or skills, or the factors associated with providing smoking cessation advice.

**Methods:**

This paper aimed to explore the knowledge and attitudes of health care providers caring for pregnant Australian Aboriginal women with regard to smoking risks and cessation; and to identify factors associated with self-reported assessment of smoking. A cross-sectional survey was undertaken with 127 staff providing antenatal care to Aboriginal women from two jurisdictions: the Northern Territory and New South Wales, Australia. Measures included respondents' estimate of the prevalence of smoking among pregnant women; optimal and actual assessment of smoking status; knowledge of risks associated with antenatal smoking; knowledge of smoking cessation; attitudes to providing cessation advice to pregnant women; and perceived barriers and motivators for cessation for pregnant women.

**Results:**

The median provider estimate of the smoking prevalence was 69% (95%CI: 60,70). The majority of respondents considered assessment of smoking status to be integral to antenatal care and a professional responsibility. Most (79%) indicated that they assess smoking status in 100% of clients. Knowledge of risks was generally good, but knowledge of cessation was poor. Factors independently associated with assessing smoking status among all women were: employer service type (*p *= 0.025); cessation knowledge score (*p *= 0.011); and disagreeing with the statement that giving advice is not worth it given the low level of success (*p *= 0.011).

**Conclusions:**

Addressing knowledge of smoking risks and cessation counselling is a priority and should improve both confidence and ability, and increase the frequency and effectiveness of counselling. The health system must provide supports to providers through appropriate policy and resourcing, to enable them to address this issue.

## Background

Reducing smoking among Australia's Indigenous people has been identified as a government priority in its efforts to "Close the Gap" between Indigenous and non-Indigenous life expectancy [1]. Approximately half the adult Indigenous population smokes, with similar rates for men and women [2]. Identified drivers of smoking among Indigenous Australians include a history of colonisation and dispossession, socio-economic disadvantage and marginalisation, acceptability and normalisation of smoking within Aboriginal social networks and the role of tobacco in social exchange [3-7].

Addressing tobacco smoking during pregnancy could bring significant health gains. Studies with pregnant Australian Indigenous women report smoking prevalence rates between 50% and 67% [8-14], approximately three times that in the non-Indigenous population [14]. Smoking during pregnancy is associated with increased risk of maternal and infant adverse outcomes. For the mother, these include higher rates of placental abruption, placenta praevia, premature labour and premature rupture of membranes [15,16]. For the baby, adverse outcomes include low birth weight, preterm birth, intra-uterine growth retardation, perinatal death and Sudden Infant Death Syndrome [15,16]. Examination of population-level data confirms these adverse outcomes among Aboriginal women [10].

Pregnancy is considered a "teachable moment"-a time when women are more motivated to modify their behaviour than at other times [17]. Numerous studies in the general population have demonstrated high spontaneous quit rates, with additional women reducing the amount smoked [17-21]. There is also some evidence to support pregnancy as a "teachable moment" among Aboriginal women. A qualitative study with pregnant Aboriginal women from Western Australia found that a few women quit smoking when they became pregnant, but the majority preferred to try to reduce the number of cigarettes smoked as quitting was too difficult and the benefits of smoking outweighed those of quitting [6]. A recent survey of pregnant Aboriginal women in New South Wales (NSW) and the Northern Territory (NT) found that 21% of women smoking at the beginning of their pregnancy reported quitting, while a further 46% reported reducing their smoking [22]. However, smoking during pregnancy among Aboriginal women remains common, perinatal data indicate low quit rates [13], and audits have revealed gaps in provision of advice [23], suggesting that more could be done to capitalise on this opportunity.

Antenatal care providers see pregnant women multiple times throughout their pregnancy, with national data indicating that 77% of Indigenous women attended for at least five antenatal visits [24]. There is strong evidence that provision of smoking cessation counselling in the antenatal period is effective [25]. In other populations, studies have confirmed that women consider provision of smoking cessation support within the antenatal clinic setting to be appropriate [26-28], that information on smoking is best provided by health professionals [29], and that this advice is an important factor in helping women quit smoking [30]. However, the approach and manner of this advice is important [31].

Providers caring for pregnant Aboriginal women are thus potentially important change agents for addressing the harms associated with smoking. Assessment of client smoking is an essential first step in providing tailored cessation advice and support. Exploring the knowledge and views of providers on the value and effectiveness of addressing smoking with pregnant Aboriginal women, and factors associated with provision of care will assist in determining the best approaches to optimising their effectiveness. To date, there have been no studies published on this topic.

## Aims

This paper explores perceptions of health care providers who provide care to pregnant Aboriginal women regarding:

a. Their estimate of the prevalence of smoking among Aboriginal women in their community;

b. Optimal and actual assessment of smoking status;

c. Their knowledge and attitudes to providing smoking cessation advice; and

d. The factors associated with self reported assessment of smoking among pregnant Aboriginal women.

## Methods

A cross-sectional survey was undertaken with staff providing antenatal care from two jurisdictions: those working in remote medical services in the NT and those providing care through the Aboriginal Maternal and Infant Health Strategy (AMIHS) in NSW. AMIHS teams are comprised of community midwives and Aboriginal Health Workers (AHWs) working together to provide outreach antenatal care for Aboriginal women in multiple sites across NSW. While both professionals work together, the midwives usually focus on the clinical aspects of antenatal care, and the AHWs address health education, community development and social needs. In the NT, staffing of remote clinics varies, with most clinics employing midwives or nurses to provide clinical aspects of antenatal care, and AHWs supporting clinical care and providing health education. Aboriginal Health Workers are a specific category of Australian health professional, working in both government health services and Aboriginal Community Controlled Health Organisations. They are usually members of the local community and work to help bridge the cultural gap between Aboriginal people and the western medical system.

A community reference group (CRG) of Aboriginal women and service providers from rural NSW was formed to guide the study and ensure it was conducted in a culturally secure manner [32]. The CRG provided input into the content and wording of the questionnaire, interpretation of findings and endorsed the reports and papers from the study.

### Recruiting participants

Staff providing antenatal care to Aboriginal women in each jurisdiction were identified by the relevant health departments. Staff were eligible if they provided antenatal care to Aboriginal women as a part of their normal role. In the NT, the DHF provided a list of staff working in remote medical services who potentially provided antenatal care (i.e. staff who were not specialists in another field such as mental health). The medical services were then contacted by the research team to establish which staff were actively involved in providing antenatal care and these staff were considered eligible for the survey. In NSW, lists of AMIHS staff were provided by the AMIHS co-ordinator in each Area Health Service. All AMIHS staff were considered eligible for the survey as this is a specific program for provision of antenatal care to Aboriginal families. Eligible staff in both jurisdictions were sent invitation letters, information sheets and self-completion questionnaires. The staff included AHWs, midwives, nurses and doctors. They were asked to complete the anonymous questionnaires and return them in pre-paid envelopes. To maximise response rates and reduce bias, reminder letters, with additional copies of the questionnaire and information sheet, were sent three weeks after the initial invitation and again a month later to all staff. Return of the questionnaire was considered to indicate implied consent. The study was conducted between September 2008 and July 2009.

### Questionnaire development and contents

#### Literature review

Concepts included in the questionnaire were derived from a review of the published literature on knowledge and attitudes of clinicians to providing advice on smoking to pregnant women [28,29,33-38] and the literature on substance use in pregnancy in general and specifically among Aboriginal peoples [3,6,9,17,26,39]. Specific questions related to knowledge of risks and attitudes towards smoking during pregnancy were adapted from a questionnaire used with pregnant Aboriginal women [40]. Additional questions derived from our research exploring the knowledge and attitudes of pregnant Aboriginal women [22] were also included.

#### Consultation with experts

The draft questionnaire was critically reviewed by several groups with a view to assessing content validity, reducing redundancy and refining the wording of questions to ensure cultural appropriateness. These groups included the CRG, the NT Department of Health and Family, and colleagues of the authors who were experienced in Aboriginal health research, tobacco control and questionnaire design. Minor revisions were made to question order and wording, with removal of some redundant questions and addition of others.

#### Pilot testing

The revised questionnaire was pilot-tested with 12 service providers, including seven midwives and five AHWs, in NSW and Western Australia, who provided additional comments. Further minor modifications were made to the wording of some questions in consultation with the CRG, prior to finalisation of the instrument.

The final questionnaire had a Flesch-Kincaid reading level of grade 9, and took approximately 15 min to complete. The items in the final questionnaire covered:

• Estimated prevalence of smoking among pregnant and non-pregnant women in the local community.

• The perceived percentage of pregnant women that should be assessed for smoking with optimal care, and the percentage actually assessed.

• Knowledge of smoking cessation and of risks associated with smoking during pregnancy.

• Attitudes to providing advice to pregnant women.

• Perceived barriers and motivators to smoking cessation for pregnant women.

• Respondents' ethnicity, gender, position and their own smoking status (current daily smoker, current occasional smoker, ex-smoker and never-smoker).

Questions related to knowledge and attitudes were presented as statements, and respondents were asked to indicate their agreement on a four-point Likert scale (strongly agree to strongly disagree), with the addition of a 'not sure' option for the knowledge questions.

### Statistical methods

The questionnaires were designed to be computer-scannable. Data were analysed using Stata 9.2. Summary statistics of respondent characteristics were obtained. Due to the non-normal distribution, respondents' estimates of prevalence of smoking among Aboriginal women are presented as medians with 95% confidence intervals. Responses to the questions about optimal and actual assessment of smoking were dichotomised into '100% of women' or 'fewer than 100% of women'. These classifications were used as they were considered to be a proxy for whether or not the respondent included assessment of smoking as a core part of routine antenatal care, or as an optional element. Responses to knowledge questions were dichotomised to 'correct' or 'incorrect', with 'not sure' classified as incorrect. The number and percentage correct are presented. Knowledge scores were generated as the sum of the correct responses for knowledge of risk and of cessation separately. Responses to attitude questions were dichotomised to 'agree' or 'disagree' and the number and percentage of respondents agreeing with the statements presented.

Univariate associations with reported assessment of smoking status were examined using the Fisher's exact chi-square test for categorical explanatory variables and the non-parametric Mann-Whitney test for continuous explanatory variables. Multivariable logistic regression was used to determine which factors were associated with self reported assessment of smoking status for all clients when adjusted for confounders. Initially all variables with a *p*-value < 0.25 in the univariate analyses and cell size ≥ 4 were included in the model, with stepwise removal of variables based on the p-value from the likelihood ratio test, with variables with a *p*-value < 0.1 retained in the model. Jurisdiction was retained in the model regardless of statistical significance as the differences in social context and service delivery between jurisdictions were considered important. Records with missing data for relevant variables were excluded from the multivariable analysis.

Based on initial information provided by the NT DHF and the NSW AMIHS program we anticipated that there would be 260 eligible service providers across both jurisdictions and with a response rate of 70% there would be 182 respondents. This would allow an estimate of the proportion of providers who assess 100% of women with 95% confidence interval within ± 7% of the point estimate if at least 60% of providers reported assessing 100% of women. It would also allow detection of differences in characteristics between providers who do and those who do not assess smoking status of 100% of pregnant women, of 22% or more with 80% power and 5% significance level.

### Ethical approval

The NT survey was approved by the Human Research Ethics Committees of the University of Newcastle and the Northern Territory Department of Human Services and Menzies School of Health Research. The NSW survey was approved by the University of Newcastle, Hunter New England and the Aboriginal Health & Medical Research Council Human Research Ethics Committees.

## Results

### Respondent characteristics

In total 184 eligible providers were identified of whom 127 (69%) responded to the survey. Of these 33 (26%) were Aboriginal, 30 (24%) were AHWs, 89 (70%) were midwives or nurses and eight were doctors (5%), with the majority (n = 96; 76%) employed in government services and the remainder in Aboriginal Community Controlled Health Services. Nineteen respondents (15%) reported being current smokers with smoking significantly more common among the AHWs (34%) than others (9.4%) (OR = 5.1; 95%CI 1.8, 14.2).

### Estimated prevalence of smoking

The median estimate of the prevalence of smoking among pregnant Aboriginal women was 69% (95%CI: 60, 70). The median clinician estimate of the prevalence of smoking among pregnant women was slightly but non-significantly lower than that for non-pregnant women in the community (75%; 95%CI: 70,80).

### Optimal and actual assessment of client smoking status

The majority of respondents (n = 103; 86%) indicated that, "with optimal care" they should know the smoking status of all their clients, with 96 (79%) indicating that they "actually ask" the smoking status of all their clients. Those who indicated that, with optimal care, they should know the smoking status of all clients were significantly more likely to claim they asked all clients (p < 0.001). Further univariate analyses revealed that current smokers, AHWs and staff employed by a community-controlled organisation were significantly less likely to report assessing the smoking status of all their clients, relative to non-smokers, other health professionals and those employed by government services respectively (Table 1). There were no differences in self-reported assessment by jurisdiction.

**Table 1:** Socio-demographic characteristics of providers by self-reported assessment of tobacco smoking (n = 122)

	Provider assesses 100% of women^a ^(n = 96)	Provider assesses fewer than 100% of women^a ^(n = 26)	*p*-value^b^
	n (%)	n (%)	
Jurisdiction			
NSW	54 (56)	11 (42)	0.269
NT	42 (44)	15 (58)	
Smoking status-current			
Smoker	11 (12)	8 (32)	0.026
Non-smoker	84 (88)	17 (68)	
Position			
AHW	16 (17)	11 (42)	0.008
Midwife/Nurse/Dr	80 (83)	15 (58)	
Service			
Community control	16 (17)	11 (42)	0.008
Government	80 (83)	15 (58)	

^a^5 missing values on question asking about actual assessment of smoking status

^b^Fisher's exact test

### Knowledge of risks associated with smoking during pregnancy and of smoking cessation

Respondents' knowledge of smoking-related risks was high (see Table 2), although the majority incorrectly indicated that smoking increased the risk of maternal pre-eclampsia. Two respondents did not agree that smoking increased the risk of low birth weight, both of whom reported not always assessing smoking status. There were no other significant differences in knowledge of risks or the total knowledge of risk score between those who did and did not report assessing smoking among all their clients.

**Table 2:** Knowledge of risks and of smoking cessation, by self-reported assessment of tobacco smoking

	Provider assesses 100% of women (n = 96)	Provider assesses fewer than 100% of women (n = 26)	
	**Correct n (%)**	**Correct n (%)**	***p*-value^a^**

**Knowledge of risk**			
Smoking during pregnancy increases the risk of:			
Miscarriage (losing the baby)	90 (94)	21 (81)	0.055
Low birth weight of baby	96 (100)	24 (92)	0.044
Breathing problems and sickness in infant	93 (97)	25 (96)	1.000
Mother having high blood pressure and increased heart rate (pre-eclampsia)^b^	3 (3)	1 (4)	1.000
Behavioural problems in childhood	55 (57)	13 (50)	0.514
Total knowledge of risk score (median (Q1, Q3))	4 (3, 4)	3.5 (3, 4)	0.247
**Knowledge of cessation**			
Nicotine replacement therapy (patches, gum etc) can help women quit	85 (89)	20 (77)	0.197
Nicotine replacement therapy shouldn't be used in pregnancy	71 (74)	11 (42)	0.004
An effective way to quit during pregnancy is to just stop altogether, right away	48 (50)	13 (50)	1.000
An effective way to quit during pregnancy is to reduce by 1 to 2 cigarettes each day^c^	26 (27)	4 (15)	0.306
Total knowledge of smoking cessation score (median(Q1, Q3))	2 (2, 3)	2 (1, 2)	0.010

^a^Fisher's exact test, except for knowledge scores which used the non-parametric Mann-Whitney test

^b^Smoking during pregnancy is not associated with increased risk of pre-eclampsia [16]

^c^Gradual reduction alone has not been shown to be an effective strategy for smoking cessation in pregnancy [25] and is not recommended in Australian national guidelines on managing smoking in pregnancy [41]

The majority of respondents considered that gradual reduction was an effective method of smoking cessation (n = 92 (75%)); while only 61 (50%) thought that stopping suddenly and completely was effective. Correct responses did not differ significantly by reported assessment of smoking status among clients (Table 2). Recognition that nicotine replacement therapy (NRT) could be used in pregnancy was significantly associated with reported assessment as was the total smoking cessation score.

### Attitudes to providing advice and perceptions of barriers and enablers to quitting

The majority of respondents agreed with statements that advising women to quit smoking was one of the main things they could do to help women have healthy babies, and that it was a service responsibility to do so, with no difference by assessment status (Table 3). There were also no differences by assessment status in the proportion indicating that helping women quit smoking made them feel proud of their role or that the Aboriginal community saw this as a priority. However, there were significant differences on several other attitudinal variables, with those who don't assess all women more likely to agree that advising women to quit smoking was not worth it given the low success rate; that they didn't have the skills; that other risks faced by women were greater; that they didn't want to push women away from antenatal care and that smoking was the woman's choice and not their responsibility. There were no significant differences by assessment status for any of the listed barriers or motivators for quitting (Table 3).

**Table 3:** Attitudes and perceived barriers and enablers for quitting, by self-reported assessment of tobacco status

	Provider assesses 100% of women (n = 96)	Provider assesses fewer than 100% of women (n = 26)	
	Strongly agree/Agree n (%)	Strongly agree/Agree n (%)	*p*-value^a^
Attitudes to advising pregnant women to quit smoking			
It is one of the main things that can be done to help women have healthy babies	91 (95)	24 (92)	0.640
Giving advice about smoking to these women is not worth it given the small level of success	4 (4)	11 (42)	< 0.001
My health service has a responsibility to encourage pregnant women to quit	94 (98)	24 (96)	0.504
I'd like to give anti-smoking advice but I don't have the skills	14 (15)	12 (48)	0.001
The harms of smoking in pregnancy are minor compared with other risks women face	8 (8)	8 (32)	0.005
I don't want to push women away from antenatal care by telling them to quit smoking	17 (18)	13 (52)	0.001
It's an individual choice. It's not up to me to tell a woman to quit smoking	4 (4)	6 (24)	0.005
Our Aboriginal community sees helping pregnant women quit smoking as a high priority	55 (58)	15 (65)	0.638
Helping women quit smoking makes me feel proud of my role	78 (82)	18 (82)	1.000
Perceived barriers and motivators to smoking cessation			
Pregnancy is a time when most women are more motivated to quit than usual	74 (77)	22 (92)	0.155
It's harder to quit during pregnancy than other times	18 (19)	6 (26)	0.403
There is no point in stopping smoking late in pregnancy	3 (3)	3 (12)	0.102
Women will try to quit for their children even if they won't try for themselves	72 (76)	18 (75)	1.000
Women who smoke cannabis find it harder to quit tobacco	66 (71)	14 (61)	0.450
Women smoke to bury their pain	59 (63)	14 (56)	0.497
Women smoke to suppress their emotions	61 (66)	16 (64)	1.000
Most women who quit in pregnancy, start again when the baby is born	67 (71)	17 (74)	1.000

^a^Fisher's exact test

N.B up to 6 missing responses for some variables

### Factors independently associated with assessment of smoking status

Logistic regression identified three variables which were independently and significantly associated with increased odds of self-reported assessment of smoking status, controlling for jurisdiction: working for a government health service; higher smoking cessation knowledge score; and disagreeing with the statement that giving advice is not worth it given the low level of success. Three other variables, although not significant at the 5% level, were significant at the 10% level and also retained in the model: smoking status; and disagreeing with the statements: I'd like to give smoking cessation advice but I don't have the skills; and I don't want to push women away from antenatal care by telling them to quit smoking (Table 4).

**Table 4:** Multivariable model of associations with assessing 100% of women for smoking status

	Odds Ratio	95% CI	p-value
Jurisdiction			
NSW	2.487	0.681, 9.080	0.168
NT *			
Smoking status			
Current smoker	0.244	0.054, 1.103	0.067
Current non-smoker *			
Service			
Community controlled	0.183	0.041, 0.811	0.025
Government *			
Smoking cessation knowledge score	1.757	0.985, 3.135	0.011
Giving advice about smoking to these women is not worth it given the small level of success			
Agree	0.095	0.015,0.590	0.011
Disagree *			
I'd like to give anti-smoking advice but I don't have the skills			
Agree	0.285	0.078, 1.041	0.057
Disagree *			
I don't want to push women away from antenatal care by telling them to quit smoking			
Agree	0.330	0.094, 1.159	0.084
Disagree *			

*reference category

## Discussion

In this study we found that better smoking cessation knowledge, a positive attitude towards providing cessation advice and being employed by a government health service were significantly associated with higher rates of self-reported assessment of smoking status while providing antenatal care to pregnant Aboriginal women. There is some indication that being a non-smoker, and disagreeing with statements expressing concern that women would be pushed away from antenatal care or about having inadequate skills were also associated with higher rates of assessment, but these relationships were not statistically significant at the 5% level.

To our knowledge, this is the first study to specifically explore the knowledge and attitudes regarding smoking among service providers caring for pregnant Aboriginal women in Australia. The study was undertaken in two jurisdictions among antenatal care providers in remote, regional and urban Australian settings. The number of eligible providers was less than anticipated and the consequent small sample may have limited our ability to identify other significant associations. However, the response rate was good and the sample is likely to represent this group of service providers reasonably well.

Consistent with studies among other antenatal care providers [28,42,43], the majority of respondents considered that assessing smoking status of all women was integral to good antenatal care and a professional and service responsibility. The majority also indicated that they do ask all women about their smoking. However, over one fifth reported not always asking all women, indicating a missed opportunity for addressing a major preventable risk factor for adverse birth outcomes. While there may be over-estimation of rates of assessment due to social desirability bias, the positive attitudes expressed by the majority of respondents are an asset with potential to be enhanced by training and skills development.

In general, knowledge of risks associated with smoking was high, particularly in relation to birth outcomes and infant illness, but not for childhood health problems. An earlier study of Australian directors of antenatal clinics identified poor specific knowledge of risks [33]. Others have reported that providers did not consider smoking a serious risk to infant health [35,44]. The uncertainty regarding risks of ongoing problems in childhood indicates gaps in knowledge and lost opportunities for conveying the true burden of antenatal smoking. As provider knowledge of risk is essential for conveying clear messages regarding risk to women, education of providers regarding the specific risks associated with smoking is essential.

Knowledge of smoking cessation was poor and inversely associated with level of assessment. Only half the respondents recognised that complete and sudden cessation was an effective quitting method; three quarters incorrectly indicated that gradual reduction was effective; and one third incorrectly indicated that NRT shouldn't be used in pregnancy despite national guidelines stating that NRT can be used in pregnancy [41]. Other studies have also identified a preference among antenatal providers for advising reduction rather than complete cessation [33-36,42,45]. Smoking cessation interventions are poorly covered in nursing curricula [46] and in training for Aboriginal Health Workers [47], which may explain the low level of knowledge and that approximately one fifth felt they didn't have the skills to provide advice. Perceived skill level is associated with provision of tobacco interventions [37,48], and lack of skills have repeatedly been identified as a barrier to smoking cessation counselling by practitioners [31,49-51]. In our study, both knowledge scores and perceptions of skills were related to level of smoking assessment, suggesting that provision of culturally appropriate, pregnancy-specific training and resources would increase confidence and skills and consequently assessment and management of antenatal smoking.

Although only a small proportion of respondents agreed that giving advice was not worth it, this perception was strongly associated with level of reported assessment, suggesting that pessimism regarding the impact of advice may contribute to non-assessment of smoking status. Pessimism about the effectiveness of interventions has been identified as a barrier to providing cessation counselling in other Australian antenatal settings [37,42,51] and internationally [38,43,44,52,53]. The poor knowledge of smoking cessation identified is likely to contribute to low efficacy of any advice provided, further contributing to a perception that advising cessation is futile. Within the context of providing care to women with multiple complex care needs with constrained resources [5,6,23], providers who anticipate low success rates may prioritise other activities which are easier to implement or have greater chance of success.

Provider smoking has been shown to be negatively associated with smoking cessation counselling [48,54] and smoking is perceived to be a barrier to providing cessation counselling among Aboriginal Health Workers [6,39,55-57]. Several studies have suggested assisting AHWs to quit in order to increase their comfort in providing cessation support [55,56]. Although only 19 (15%) of the respondents smoked, provision of smoking cessation support to those who do is likely to be beneficial for the individuals, enhance their willingness to provide cessation supports, and give them personal experience in quitting.

One quarter of the respondents indicated concern that providing advice might push women away and this was associated with lower smoking assessment, although this was not significant at the 5% level in the multivariable analysis. In a Western Australian study of smoking during pregnancy, AHWs expressed discomfort about raising smoking as they wished to maintain positive relationships with women [6]. Similar concerns have been identified in other studies with AHWs, although they report feeling more comfortable discussing smoking with pregnant smokers than with other smokers [39,56,57]. In other antenatal settings, a perception that clients are not interested or do not expect advice, has been identified as a barrier [37,52], and midwives have expressed concern about potentially damaging their relationship with women if they address their smoking [28,31,50]. By contrast, women consider provision of smoking cessation advice within antenatal care to be acceptable [26], and state that it doesn't affect their relationships with their midwives [27]. However, the manner of providing care is important, and should not be authoritarian [28,50,54,58,59]. Greater community and provider understanding of the real risks of smoking and benefits of cessation may increase community support and help providers feel more comfortable addressing smoking.

Limitations to this research should be considered in interpreting the findings. In addition to the small sample size and potential social desirability bias mentioned above, the cross-sectional nature of the survey prevents assessment of causality in the relationship between assessment and the knowledge and attitudinal variables. Factors other than the knowledge and attitudinal variables included in our study may be determining respondents smoking cessation activities, and the reported attitudes may then reflect a rationalisation on the part of respondents to justify their behaviour. Trials of interventions that aim to address knowledge and attitudes would be beneficial in assessing this. A further limitation is that the study did not assess the amount or type of advice that the clinicians provide to women.

## Conclusions

This study has identified factors constraining the provision of evidence-based antenatal care in relation to tobacco use, but has also found strengths on which to build. The majority of providers recognised that smoking increased the risk of adverse outcomes, considered that giving cessation advice was important, and believed that providing advice was their responsibility. The majority reported assessing all women for smoking and saw it as part of optimal antenatal care.

The poor knowledge of providers regarding smoking cessation reinforces the call from others for development of culturally appropriate training and resources for providers caring for Aboriginal peoples [5,47,55,60], including those specific to pregnancy [6,61]. Addressing knowledge of risks and smoking cessation counselling among antenatal providers is a priority and should improve both confidence and ability, and increase the frequency and effectiveness of counselling. Programs designed to support pregnant women to quit smoking need to address the many drivers of smoking, including high levels of stress and disadvantage, and social norms of smoking [4-7]. Additionally, the health system must provide supports to providers through appropriate policy and resourcing, to enable them to address this issue. Recent government initiatives in Indigenous smoking are likely to raise recognition of the importance of addressing smoking at every opportunity and should be accompanied by broader efforts to address Indigenous disadvantage.

## Abbreviations

AHW: Aboriginal health worker; AMIHS: Aboriginal maternal and infant health strategy; CRG: Community reference group; NSW: New South Wales; NT: Northern territory.

## Competing interests

The authors declare that they have no competing interests.

## Authors' contributions

MP and RSF conceived of the study and developed the study design, data collection instruments and procedures. MP collected the data. Statistical analysis was carried out by MP, CDE and RSF. All authors participated in data interpretation. MP led manuscript preparation. All authors revised the manuscript critically and approved the final version.

## Pre-publication history

The pre-publication history for this paper can be accessed here:

http://www.biomedcentral.com/1471-2458/12/165/prepub
